# Exploring the Differentiation of Self-Concepts in the Physical and Virtual Worlds Using Euclidean Distance Analysis and Its Relationship With Digitalization and Mental Health Among Young People: Cross-Sectional Study

**DOI:** 10.2196/60747

**Published:** 2025-01-31

**Authors:** Kai Tai Chan, Christy LM Hui, Charlton Cheung, Yi Nam Suen, Stephanie Ming Yin Wong, Corine SM Wong, Bosco PH Kam, Eric Yu Hai Chen

**Affiliations:** 1 Department of Psychiatry Chinese University of Hong Kong Hong Kong China (Hong Kong); 2 Department of Psychiatry University of Hong Kong Hong Kong China (Hong Kong); 3 School of Nursing Li Ka Shing Faculty of Medicine University of Hong Kong Hong Kong China (Hong Kong); 4 Department of Social Work and Social Administration University of Hong Kong Hong Kong China (Hong Kong); 5 School of Public Health, Li Ka Shing Faculty of Medicine University of Hong Kong Hong Kong China (Hong Kong); 6 Orygen Center for Youth Mental Health University of Melbourne Melbourne Australia

**Keywords:** digitalization, self, identity, psychiatric symptomatology, youth mental health, Euclidean distance analysis, self-differentiation, smartphone addiction, personal attributes

## Abstract

**Background:**

Increasing observation and evidence suggest that the process of digitalization could have profound impact to the development of human mind and self, with potential mental health consequences. Self-differentiation is important in human identity and self-concept formation, which is believed to be involved in the process of digitalization.

**Objective:**

This study aimed to investigate the relationship between digitalization and personal attributes in the actual selves in the physical and virtual worlds.

**Methods:**

A community cohort of 397 participants aged 15 to 24 years old was recruited consecutively over about 3 months. Assessment was conducted upon the indicators of digitalization (smartphone use time, leisure online time, and age of first smartphone ownership), smartphone addiction, 14 selected personal attributes in the actual selves in the physical and virtual worlds, psychiatric symptomatology and personality traits. Euclidean distance analysis between the personal attributes in the actual selves in the physical and virtual worlds for the similarities of the 2 selves was performed in the analysis.

**Results:**

The current primary findings are the negative correlations between the similarity of the personal attributes in the physical actual self and virtual actual self, and smartphone use time, smartphone addiction as well as anxiety symptomatology respectively (*P*<.05 to *P*<.01).

**Conclusions:**

The current findings provide empirical evidence for the importance of maintaining a congruent self across the physical and virtual worlds, regulating smartphone use time, preventing smartphone addiction, and safeguarding mental health.

## Introduction

### Background

The internet and digital devices have been infiltrating rapidly into the world, human society and culture as well as everyday living, especially after the global popularization of the smartphone, which is a personalized communication tool with full computer functions and 24/7 accessibility. Across the millennium, there has been postulation of “Digital Natives” as well as “Digital Immigrants,” depicting the potential influence of digitalization across the generations [1]. In recent decades, it has witnessed growing evidence of the impact of digitalization on the human brain, mind, cognition, and behavior [2]. Such observations and neurobiological findings imply that our brain, mind, and self can be modified in the process of digitalization in a direct and profound way. To delineate the active and overall process of digitalization upon the human self, there is a postulation of a “Digitalized Self” [3].

### Concepts and Theories of Self, Identities, and Personal Attributes

There has been ongoing development of different conceptualization and theorization of the self according to contemporary knowledge and evidence. Based on the classic work by James [4], recent views tend to regard the self as both a unity and a multiplicity [5,6]. Subsequent works have been further exploring the properties of the self, such as structural properties of the self and their potential relationship with psychological well-being, as well as separate self-schemas [7]. Among various theories about the differentiation of the self according to different conditions and contexts, there comes the self-concept differentiation [8], which involves adaptation to the different requirements of different social roles by specialization and fragmentation [9]. Regarding different types of self-states, under the self-discrepancy theory [10], there are “actual self,” “ideal self,” and “ought self.” Relevant to mental health, potential mismatches of these different kinds of self-states from the perspectives of oneself and others would be associated with different emotional states.

Concerning identities and personal attributes in different roles, for social identities, they are representation of self in social settings while for personal attributes, they are features or qualities of individuals. According to the integration model, they are organized in terms of several clusters, each of which combines identities and attributes [11]. According to the segregation model, personal attributes are organized under 2 major clusters of social identities, namely the collective self and the private self [12].

Based on these various theories, the development of self can be regarded as a continuous interaction of one’s mind with the environment at different levels and with different facets according to different situations and roles, through the processes of differentiation and integration, leading to different selves with specific identities and personal attributes for that particular individual. Hence, the similarities and differences between different selves of an individual might be a reflection of the processes of self-development, which would be related to various mental health and behavioral outcomes [13].

### Relationship Between Digitalization, Self, and Personal Attributes

It has been theorized under different perspectives that construction of online self-presentation and online identities would be subject to the specific aspects of self and the specific characteristics of the virtual world as well as their interaction, which would involve different mechanisms, functions, and outcomes [14,15]. To explore the evidence timely, recent reviews [16-18] and researches [19-22] have explored the issues of online presentation, self-discrepancy, as well as online identity reconstruction in the social media and virtual world with reference to the impact in human and social development as well as well-being. Preliminary findings have been found in different aspects such as self-esteem, personalities, and behaviors, which would in turn have fundamental implications to humankind adaptation in the changing environment as well as human development and mental health. As the development of research in the area is at an early stage, the knowledge and evidence so far are diverse and fragmented. In order to integrate various theoretical frameworks into existing evidence for further exploration, based on the concept of “Digitalized Self” [3], we postulated there would be a dual process involving both transition of the physical self into the virtual self as well as the indigenous birth virtual self when the boundary between the physical and virtual worlds become blurred. So far, we cannot find a study about the differentiation of the physical self and virtual self in the physical and virtual worlds which takes these 2 environments with associated interactions into account. That is the purpose of the current study to timely fill in the current knowledge gap. In this study, we chose personal attributes related to social context among the different components of the self for examination, which can highlight the interactive processes between humans and social environment in shaping the self.

**Figure 1:**
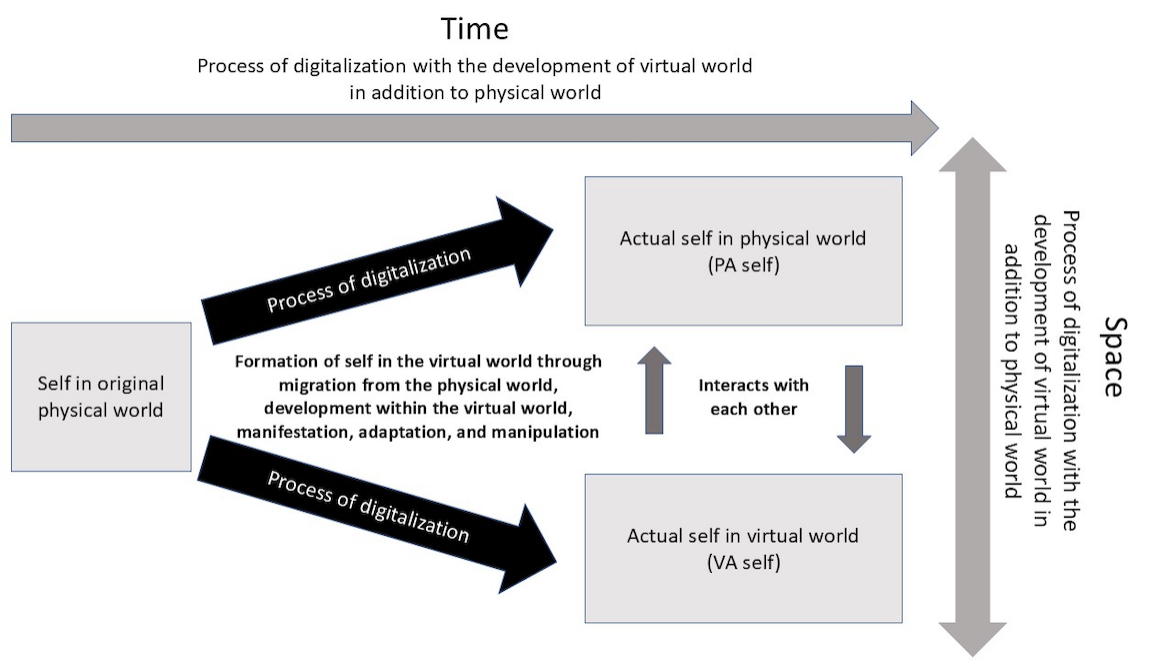
Differentiation of self in the process of digitalization: transition and interaction between physical actual self and virtual actual self across physical world and virtual world. PA: physical actual; VA: virtual actual.

Based on the postulated processes in the formation of “Digitalized Self” [3], it is postulated that the self in the original physical world would develop into actual selves in the physical and virtual worlds through self-migration from the physical world and formation of additional self in the virtual world, leading to the similarities and differences between the actual selves in the physical and virtual worlds (Figure 1). As far as the formation of personal attributes is concerned, while it can be the direct shaping, modulation and manifestation of the personal attributes in the physical world and virtual world in the process of digitalization, it can also be the adaptation of a particular personal attribute to that particular environment which determines its level of expression in the physical world and virtual world. In the end, the personal attributes in the physical and virtual worlds could also interact with each other, further shaping each other.

### Study Conceptualization and Hypothesis

Based on the aforementioned conceptualization and theorization, in this study, we aim at exploring the relationship between digitalization-related measurements (smartphone use time, leisure online time, age of first smartphone ownership, and smartphone addiction), the similarity of personal attributes in the actual selves in the physical world and virtual world as well as mental health outcomes. We would further discuss the postulation about the underlying mechanisms and development process of the impact of digitalization on the differentiation of personal attributes in the physical and virtual worlds. The primary hypothesis of our study is that there would be a correlation of the similarity of the 14 personal attributes in the physical actual (PA) self and the virtual actual (VA) self with different measurements of digitalization (including smartphone addiction) and psychiatric symptomatology.

## Methods

### Study Design

This study is a cross-sectional study, which is a consecutive cohort within the Hong Kong Youth Epidemiological Study of Mental Health (HK-YES), the epidemiological study for young people in Hong Kong with a sample size of 3340 [23-25]. A random stratified method was adopted to select participants with the addresses provided by the Census and Statistics Department of the Hong Kong Special Administrative Region (HKSAR), based on the geographical location and type of quarters. Further details of the HK-YES have been reported elsewhere [23-25].

### Settings

As a part of a large epidemiological study in Hong Kong, the setting of this study is in the community, which was conducted upon a consecutive cohort recruited between June 10, 2021, and August 30, 2021. As a community sample, the exposure of the participants would be the influence of digitalization in the community.

### Data Collection

All participants provided written informed consent before enrollment in the study. Informed consents from parents or legal guardians were obtained for participants aged 18 years or younger. Afterwards, the participants were interviewed by a trained research assistant who would provide clarity and guidance on answering questions. Interviews were conducted in both online and offline formats due to the social distancing policies during the COVID-19 pandemic.

### Participants

The inclusion and exclusion criteria are presented in Textbox 1. A total of 397 participants were recruited during the study period.

**Participant inclusion and exclusion criteria.**

**Inclusion criteria:**

- Aged between 15 to 24 years old at the time of recruitment.
- Ability to give consent for participation.
- Hong Kong resident.

**Exclusion criteria:**

- Not having Chinese as a first language.

### Variables and Measurements

#### Personality Attributes in Selves

To measure different types of personal attributes, 14 personal attributes related to different identities were selected from a list of personal attributes in a previous study [26]. The method in measuring a person’s self-concept has been described [27], while specific personal attributes were chosen to reflect changes in self-concept [26]. The list includes the dimensions of different roles of oneself and others in a relevant and meaningful way validated by the idiographic and hierarchical classes analysis model. The 14 personal attributes include “Aggressive,” “Calculating,” “Capable,” “Compromising,” “Deceiving,” “Excluding,” “Helpful,” “Gossiping,” “Hostile,” “Investigative,” “Manipulative,” “Powerful,” “Trustworthy,” and “Vengeful” [26]. It covers the nonprosocial, prosocial, and competence personal attributes, which would develop under different self-identities in the physical and virtual worlds in order to survive, socialize, and achieve.

#### Measurements of Digitalization

The degrees of digitalization in the environment were captured by smartphone use time (in terms of hours per day), leisure online time (in terms of hours per day), and age of first smartphone ownership (in terms of years). In addition, smartphone addiction is also measured to reflect the influence of digitalization within individuals where the pathological process and individual vulnerabilities would also be involved. For smartphone addiction, the Revised Chen Internet Addiction Scale (CIAS-R), a 26-item self-reported questionnaire on a 4-point Likert scale [28], measures one’s level of smartphone addiction on 5 different factors. They are withdrawal symptoms, compulsive use, tolerance, interpersonal and health-related problems, and time management problems. All 26 items are included to create a total CIAS-R score. Psychometrics of the CIAS-R were previously examined in the Hong Kong population [29]. A higher CIAS-R score indicates a higher severity of internet addiction. Based on previous studies, a diagnostic cutoff point of 67/68 would be adopted, indicating that individuals scoring 68 or above in CIAS-R would be diagnosed as internet addiction [30]. In the current study, the CIAS-R was used to capture smartphone addiction by referring to smartphone use behavior.

#### Other Variables and Measurements

Demographics information, symptomatology of stress and common emotional issues as well as personality traits were also examined. Symptomatology of stress and common mental health problems was measured by Depression Anxiety Stress Scale [31,32]. General personality traits were measured by the Big Five Inventory, which consists of 44 items rated on a 5-point Likert scale [33]. Both of these scales have been validated and used in youth samples in Hong Kong [34,35].

#### Calculation of Euclidean Distances

In this study, to measure the difference of formation of personal attributes in the physical and virtual worlds, the calculation of their Euclidean distance [36] was done by squaring the differences between respective code values in the physical world (q) and virtual world (p) for each of the 14 personal attributes (codes 1 for presence of a particular personal attribute and 0 for absence of a particular personal attribute) followed by taking the square roots of the final summation. The formula is summarized as follows (n=14):



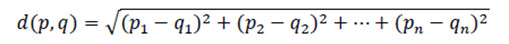



#### Biases

Potential biases in the primary epidemiological study were minimized by random stratified sampling, which was addressed in the paper of the primary study. For current study, our bias was minimized by consecutive sampling over a period of almost 3 months in the primary study.

#### Study Size

As this is a pilot study of its kind, we did not have previous data to estimate the sample size. The current sample size was estimated according to the number of variables.

#### Statistical Methods

Data analyses were conducted using SPSS (version 27.0; IBM Corp), with statistical significance at the *P*<.05 level. The assumptions of the relevant data are that they are in normal distribution and variances between different variables do not differ from each other. To adjust for multiple comparison problems, Benjamini–Hochberg procedure was performed for all correlation analyses to control for the false discovery rate (FDR). Likewise, Bonferroni correction was performed for all independent *t* test analyses to adjust for multiple comparison problems.

#### Data Analyses

First, correlations between smartphone addiction, smartphone use time, leisure online time, age of first smartphone ownership, personality traits, and symptomatology of stress and common emotional issues as well as the Euclidean distance between the 14 personal attributes in the PA self and the VA self (PA/VA distance) were examined. Second, partial correlations between constructs, where potential confounders were held constant, were done to ascertain the exact relationship between digitalization and mental health with the Euclidean distance between the 14 personal attributes in the PA self and the VA self (PA/VA distance).

### Ethical Considerations

Approval from the institutional review board of the University of Hong Kong/ Hospital Authority Hong Kong West Cluster (UW 19-017) was obtained.

## Results

### Overview

A consecutive cohort of 397 eligible participants participated in this current project, with 24 having missing data. The missing data were excluded during analysis.

### Descriptive Data and Comparisons of Demographics, Indicators of Digitalization, and Smartphone Addiction

Table 1 shows descriptive statistics for age, sex, smartphone use time, leisure online time, age of first smartphone ownership, and smartphone addiction as an overview.

**Table 1:** Basic information of age, sex, smartphone use time, leisure online time, age of first smartphone ownership, and smartphone addiction both for all and between sex groups.

Variables	All participants (N=397), mean (SD)	Sex	
		Male (n=183), mean (SD)	Female (n=214), mean (SD)
Age (years)	19.43 (2.77)	19.41 (2.76)	19.45 (2.78)
Smartphone use time (hours per day)	5.90 (3.27)	5.59 (3.41)	6.17 (3.14)
Leisure online time (hours per day)	6.05 (3.26)	6.67 (3.51)	5.53 (2.93)
Age of first smartphone ownership (years) (n=392)	11.91 (2.28)	12.06 (2.36)	11.78 (2.22)
CIAS-R^a^ score (smartphone addiction) (n=395)	59.55 (11.66)	57.46 (11.31)	61.36 (11.75)

^a^CIAS-R: Revised Chen Internet Addiction Scale.

For the bivariate correlation between age, smartphone use time, online leisure time, age of first smartphone ownership, and smartphone addiction, smartphone use time is significantly positively correlated with leisure online time (*r*_395_=0.439, *P*<.001). Smartphone addiction score CIAS-R is significantly positively correlated with smartphone use time (*r*_393_=0.261, *P*<.001). Age of first smartphone ownership is significantly positively correlated with age (*r*_390_=0.560, *P*<.001). Because of significant correlation between age and age of smartphone ownership as well as the potential generation-related cohort effect, a partial correlation analysis was done for correlation analysis of age of first smartphone ownership with other variables to control for the effect of age. Age of first smartphone ownership (controlled for effect of age) is significantly negatively correlated with smartphone use time (*r*_390_=–0.151, *P*<.01), and significantly negatively correlated with smartphone addiction (*r*_390_=–0.114, *P*=.03).

Furthermore, an independent *t* test was done for the difference in smartphone use time, leisure online time, age of first smartphone ownership, and CIAS-R between male participants and female participants, using Bonferroni method with adjusted *P* value of .02. Independent *t* test suggested that female participants (mean 61.36, SD 11.75) had higher smartphone addiction scores than male participants (mean 57.46, SD 11.31; t_393_=3.36; *P*<.01), but female participants (mean 5.53, SD 2.93) had lower leisure online time than male participants (mean 6.67, SD 3.51; t_395_=3.51; *P*<.001).

The above basic information of the current cohort would also be reported in other research papers of the same series by the first author.

### Distribution of Euclidean Distance Between Personal Attributes in the Physical Actual and Virtual Actual Selves

For the Euclidean distance between personal attributes in the PA and VA selves (PA/VA distance), the mean is 1.716 (SD 0.761), the median is 1.732 (range 0-3.317).

### Correlations of Demographics, Indicators of Digitalization, and Smartphone Addiction With Euclidean Distance Between Personal Attributes in Physical Actual and Virtual Actual Selves

In Table 2, the Euclidean distance between personal attributes in the PA and VA selves (PA/VA distance) is significantly positively correlated with age (*r*_392_=0.111, *P*=.03), significantly positively correlated with smartphone use time (*r*_392_=0.116, *P*=.02), significantly positively correlated with smartphone addiction (*r*_390_=0.233, *P*<.001), significantly positively correlated with stress symptomatology (*r*_390_=0.186, *P*<.001), significantly positively correlated with anxiety symptomatology (*r*_390_=0.235, *P*<.001), and significantly positively correlated with depressive symptomatology (*r*_390_=0.165, *P*<.01). All correlations remain statistically significant after the FDR correction.

**Table 2:** Correlation matrix of Euclidean distance of personal attributes between PA/VA selves with demographics, indicators of digitalization, smartphone addiction, and psychiatric symptomatology.

Variables	Euclidian distance
Age (n=394)	.111^a^
Smartphone use time (n=394)	.116^a^
Leisure online time (n=394)	.045
Age of first smartphone ownership (Controlled for age) (n=389)	–.008
CIAS-R^b^ (Smartphone addiction) (n=392)	.233^c^
DASS-S^d^ (n=392)	.186^c^
DASS-A^e^ (n=392)	.235^c^
DASS-D^f^ (n=392)	.165^c^

^a^*P*<.05, 2-tailed (remaining significant after FDR correction).

^b^CIAS-R: Revised Chen Internet Addiction Scale.

^c^*P*<.01, 2-tailed (remaining significant after FDR correction).

^d^DASS-S: Depression Anxiety Stress Scale-Stress.

^e^DASS-A: Depression Anxiety Stress Scale-Anxiety.

^f^DASS-D: Depression Anxiety Stress Scale-Depression.

In Table 3, for the bivariate correlation between the Euclidean distance of personal attributes between the PA and VA selves (PA/VA distance) and Big Five personality traits, the Euclidean distance is significantly negatively correlated with agreeableness (*r*_392_=–0.284, *P*<.001), and significantly positively correlated with neuroticism (*r*_392_=0.228, *P*<.001). Both correlations were statistically significant after the FDR correction.

**Table 3:** Correlation matrix of Euclidean distance of personal attributes between physical actual and virtual actual selves and Big Five Personality Traits.

Variables	Euclidean distance
Openness (n=394)	.003
Conscientiousness (n=394)	–.058
Extraversion (n=394)	–.040
Agreeableness (n=394)	–.284^a^
Neuroticism (n=394)	.228^a^

^a^*P*<.01, 2-tailed (remaining significant after false discovery rate correction).

### Controlling for Potential Confounders in Partial Correlation

In Table 4, partial correlation analyses have been done in view of the intercorrelations between demographics, indicators of digitalization, smartphone addiction, Big Five personality traits, and psychiatric symptomatology, and hence potential confounding effects. Controlling for effect of age, sex, age of first smartphone ownership, Big Five personality traits, and psychiatric symptomatology, smartphone use time and CIAS-R are still significantly positively correlated with the Euclidean distance between the PA self and the VA self (PA/VA distance; *r*_359_=0.121, *P*=.02 and *r*_359_=0.145, *P*<.01, respectively). Controlling for age, sex, age of first smartphone ownership, Big Five personality traits, and smartphone addiction, anxiety symptomatology remains significantly positively correlated with the Euclidean distance between the PA self and the VA self (PA/VA distance; *r*_361_=0.146, *P*<.01).

**Table 4:** Partial correlations of indicators of digitalization, smartphone addiction and psychiatric symptomatology with Euclidean distance of personal attributes between physical actual self and virtual actual self.

Variables	Euclidean distance
Smartphone use time (n=372)	.121^a^
CIAS-R^b^ (smartphone addiction; n=372)	.145^c^
DASS-S^d^ (n=372)	.045
DASS-A^e^ (n=372)	.146^c^
DASS-D^f^ (n=372)	.075

^a^*P*<.05, 2-tailed (remaining significant after FDR correction).

^b^CIAS-R: Revised Chen Internet Addiction Scale.

^c^*P*<.01, 2-tailed (remaining significant after FDR correction).

^d^DASS-S: Depression Anxiety Stress Scale-Stress.

^e^DASS-A: Depression Anxiety Stress Scale-Anxiety.

^f^DASS-D: Depression Anxiety Stress Scale-Depression.

## Discussion

### Principal Findings

The primary findings of the current study are that the Euclidean distance of the 14 personal attributes between the PA self and VA self (PA/VA distance) correlates positively with smartphone use time, smartphone addiction and anxiety symptomatology respectively, even after controlling for the effects of potential confounders. That would mean that there is a negative correlation between the similarity of 14 personal attributes between the PA self and VA self, and smartphone use time, smartphone addiction as well as anxiety symptomatology respectively. In general, it would be rated as small effect size under usual statistical standards [37], but the current primary findings of positive correlations (0.121 to 0.146) would be considered as significant in view of the abstract and conceptual nature of the parameters.

Based upon these findings of correlations, we further attempt to infer the causality between the similarity of the 14 personal attributes between PA self and VA self, and smartphone use time, smartphone addiction, and anxiety symptomatology respectively. For the first possible direction, people with higher similarity of personal attributes between the PA self and the VA self could cause lower degree of smartphone use time, smartphone addiction, and anxiety symptomatology, as it would induce less cognitive dissonance [38] and self-incongruence [39] of actual selves between the physical and virtual worlds. In turn, it would be related to less maladaptive behavior such as excessive smartphone use, smartphone addiction, and psychological distress such as anxiety symptomatology.

On the contrary, people with lower similarity of personal attributes in the PA self and the VA self might cause higher degree of smartphone use time, smartphone addiction and anxiety symptomatology, as it would induce more cognitive dissonance and self-incongruence, which would be related to more maladaptive behavior and psychological distress. As a matter of fact, it is generally agreed that self-congruence is associated with mental well-being [40]. On the other hand, noncongruence of the self can be associated with negative mental health outcomes such as emotional and behavioral problems.

For the second possible direction of causality, it can be in the opposite direction in that excessive smartphone use and smartphone addiction might induce a higher differential migration of original personal attributes from the physical world to the virtual world as well as a more active birth and different formation of personal attributes in the virtual world, which might be advantageous for the adaptation and survival of oneself to the different environments and requirements in the physical and virtual worlds. The pathological process within vulnerable individuals in smartphone addiction in addition to the process of digitalization might further enhance this process of differentiation of the self.

For the negative correlation between anxiety symptomatology and the similarity of the PA self and VA self, it is possible that the feature of perceived threat in anxiety might induce a more active formation of different personal attributes in different environments to cope with the stressors in different environments with different requirements in the physical and virtual worlds.

We would believe the aforementioned mechanisms involved in these 2 opposite directions are different operating to different extent in different youth with different vulnerabilities, which eventually can be interrelated in vicious cycles. These explanations would lead to the importance of maintaining a congruent self between the physical and virtual worlds to reduce cognitive dissonance and self-incongruence and hence less maladaptive online behavior such as smartphone addiction and psychiatric symptomatology. On the other hand, regulating smartphone use time, prevention of smartphone addiction and safeguarding mental health would maintain self-congruence between the physical and virtual worlds.

For the third possibility, a confounding third factor could influence smartphone use time, smartphone addiction and anxiety symptomatology, and the similarity of the personal attributes in the VA self to the PA self at the same time. For example, personality traits can affect them simultaneously. However, this is not likely as these factors have been controlled appropriately in the analysis by partial correlation.

### Strengths and Limitations

The strength of the study is the quantitative capture of the abstract construct of personal attributes by using a set of researched personal attributes from a previous relevant study. The methodology is based on theory-informed construct. We have also performed partial correlation for our primary results to control for the potential effect of potential confounders to make our conclusion valid.

To capture the degree of digitalization in the environment in a quantitative and comprehensive way, we measured smartphone use time, leisure online time, age of first smartphone ownership and smartphone addiction. As there is a positive correlation of the age with the age of first smartphone ownership, the effect of age has been removed in the analysis of age of first smartphone ownership to improve its accuracy to reflect digital nativity.

Another strength of our study is the innovative and comprehensive measurement of personal attributes in both the physical and virtual worlds, which can capture and compare the formation and development of different selves of personal attributes in the original physical world and the emerging virtual world in order to postulate the possible processes and mechanisms.

The limitations of the study would include the subjective self-reporting of abstract personal features, which would be subject to the recollection, matching and projection of personal features based on values and concepts of individuals and would affect the validity of measurements. To address this issue, we have trained research assistants to explain the meanings of research questions for standardized clarification. For the interpretation of results about smartphone addiction, we need to take into account the underlying pathological component and individual vulnerabilities in addition to the process of digitalization within individuals. We have adopted the use of Euclidean distance [41] between the personal attributes in actual self in physical and virtual worlds as a quantitative comparison of the similarity of the complex constructs of personal attributes, which might not be able to fully reflect and compare the exact psychometric properties for such constructs. Furthermore, we have only included the social and competence aspects of personal attributes, which can limit the generalizability of the results.

In addition, as our participants were aged between 15 and 24 years, it would be necessary to conduct similar studies in the older age group of digital immigrants and compare them to delineate the effect of generations. In view of the nature of the cross-sectional study, the causal relationship between digitalization with the similarity of 14 personal attributes between the PA self and VA self cannot be ascertained, which would warrant longitudinal studies to explore.

### Conclusion

Personal attributes are individual traits and assets with different expression and manifestation in different self-identities in different settings and environments, the congruence of which would be essential to mental well-being and mental health. The current primary findings are the negative correlations between the similarity of the personal attributes in the PA self and VA self, and smartphone use time, smartphone addiction as well as anxiety symptomatology respectively. Hence, the key message to the public is the importance of maintaining a congruent self across the physical and virtual worlds, regulating smartphone use time, preventing smartphone addiction, and safeguarding mental health before possible irreversible changes. That can be achieved by advocating healthy and regulated smartphone use, keeping a clear boundary between humans and digital devices and items as a tool, advocating youth mental health as well as advocating self-congruence across the physical and virtual worlds, especially for the young generations. Of course, this should be coupled by enhancing age-appropriate digital literacy and safety in the virtual world, as different manifestations of selves in the physical and virtual worlds might have served a self-protective function for defense and survival in the midst of the 2 changing worlds. However, it is easier said than done, which would require further discussion and timely consensus of different stakeholders including community people, young people, Information Technology entrepreneurs, scientists and researchers, academics, clinicians as well as mental health professionals.

## Abbreviations

CIAS-R: Revised Chen Internet Addiction Scale
FDR: false discovery rate
HK-YES: Hong Kong Youth Epidemiological Study
HKSAR: Hong Kong Special Administrative Region
PA: physical actual
VA: virtual actual
